# Norovirus Capsid Protein-Derived Nanoparticles and Polymers as Versatile Platforms for Antigen Presentation and Vaccine Development

**DOI:** 10.3390/pharmaceutics11090472

**Published:** 2019-09-12

**Authors:** Ming Tan, Xi Jiang

**Affiliations:** 1 Division of Infectious Diseases, Cincinnati Children’s Hospital Medical Center, Cincinnati, OH 45229, USA; 2 Department of Pediatrics, University of Cincinnati College of Medicine, Cincinnati, OH 45229, USA

**Keywords:** nanoparticle, vaccine platform, P particle, S particle, protein polymer, subviral particle, subunit vaccine, norovirus, rotavirus, hepatitis E virus, astrovirus

## Abstract

Major viral structural proteins interact homotypically and/or heterotypically, self-assembling into polyvalent viral capsids that usually elicit strong host immune responses. By taking advantage of such intrinsic features of norovirus capsids, two subviral nanoparticles, 60-valent S_60_ and 24-valent P_24_ nanoparticles, as well as various polymers, have been generated through bioengineering norovirus capsid shell (S) and protruding (P) domains, respectively. These nanoparticles and polymers are easily produced, highly stable, and extremely immunogenic, making them ideal vaccine candidates against noroviruses. In addition, they serve as multifunctional platforms to display foreign antigens, self-assembling into chimeric nanoparticles or polymers as vaccines against different pathogens and illnesses. Several chimeric S_60_ and P_24_ nanoparticles, as well as P domain-derived polymers, carrying different foreign antigens, have been created and demonstrated to be promising vaccine candidates against corresponding pathogens in preclinical animal studies, warranting their further development into useful vaccines.

## 1. Introduction

Noroviruses, members of the *Norovirus* genus in the family *Caliciviridae*, are the most important viral pathogens causing acute gastroenteritis, affecting millions of people of all ages worldwide. The viruses are highly contagious, often leading to large outbreaks of acute gastroenteritis in closed and semiclosed settings, such as cruise ships, large military battle ships, schools, hospitals, and nursing homes [1]. In the United States, noroviruses cause around 21 million cases of acute gastroenteritis each year and are responsible for about 90% of all nonbacterial outbreaks of gastroenteritis [2]. On a worldwide basis, norovirus infections claim approximately 218,000 lives annually, with significant morbidity and economic losses [3,4,5]. However, there are no commercial vaccines or antivirals against noroviruses currently, although recombinant virus-like particle (VLP)-based vaccines are in the clinical trial stage [2,6]. Thus, noroviruses remain a major threat to public health.

**Figure 1:**
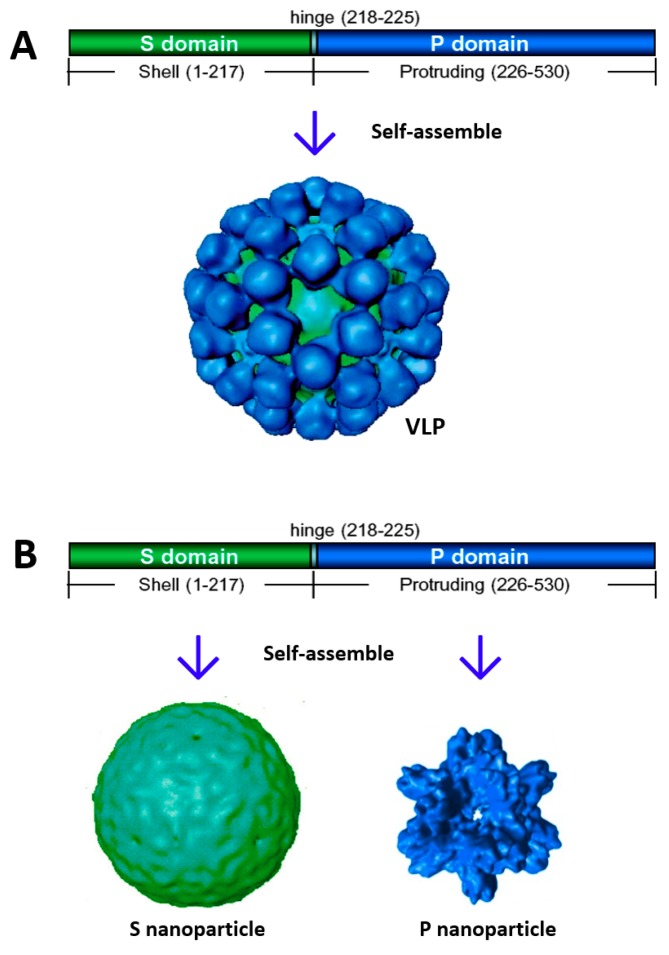
Lineage structures of norovirus capsid protein or viral protein 1 (VP1) and various nanoparticles derived from full-length or truncated VP1. The N-terminal shell (S) (green) and the C-terminal protruding (P) (dark blue) domains with a short flexible hinge (light blue) in between (with amino acid numbers based on GI.1 Norwalk virus VP1) are shown. (**A**) Production of full-length norovirus VP1s via a eukaryotic expression system self-assembles into virus-like particles (VLPs). (**B**) Production of the S or P domain via the *Escherichia coli* expression system self-assembles into S or P nanoparticles.

Noroviruses are single-stranded, positive-sense RNA viruses, each containing an RNA genome about 7.5 kilo-nucleotides in length that is composed of three open reading frames (ROFs) [1]. ORF 1 codes a large polyprotein that is post-translationally cleaved into six nonstructural viral proteins (VPs). ORF 2 and 3 code VP1 and VP2, which are the major and minor structural proteins of the noroviral capsid, respectively. Heterologous expression of norovirus VP1 via a eukaryotic system self-assembles into empty norovirus-like particles (Figure 1A) that are structurally and antigenically similar to the authentic viral capsid. A recent study on feline calicivirus (FCV) showed that VP2s form a portal-like channel through the capsid after virus–receptor engagement for delivering viral genomes into the cytosol of host cells [7]. Noroviruses have not grown efficiently in a conventional cell culture system so far, and thus norovirus VLPs offer an important norovirus research model and an excellent vaccine candidate against noroviruses. 

The structures of norovirus VLPs have been thoroughly studied via electron cryo-microscopy (cryo-EM) [8,9] and X-ray crystallography [10]. Noroviral capsids are composed of 180 VP1s that organize in a *T* = 3 icosahedral symmetry. Structurally, the capsid can be divided into two concentric layers: the interior layer is formed by the continual shell, while the outer layer is formed by 90 separate protrusions extending from the inner shell. Accordingly, each VP1 is divided into the N-terminal shell (S) domain and the C-terminal protruding (P) domain, which are linked by a short, flexible hinge [10]. The icosahedral shell is built by 180 S domains with a central lumen about 23 nm in diameter, providing a basic scaffold of the norovirus capsid. The P domains constitute 90 dimeric protrusions extending outward from the inner shell, forming the exterior surface of the capsid.

The protrusions of norovirus capsid interact with viral glycan receptors for attachment to host cells to initiate an infection (reviewed in References [11,12,13,14,15]). The crystal structures of norovirus VLPs indicate that the S domains interact homotypically [10], driving self-formation into norovirus capsids. The P domains also exhibit strong homotypic interactions, forming dimeric protrusions to stabilize the viral capsid [10,16]. In addition, the P domains also exhibit oligomeric interactions at the five-fold axis [10] (Figure 1A), suggesting that the P domains can also form oligomers or polymers in addition to the P dimers. These structural properties of norovirus capsids have been used for design and engineering of full-length and truncated norovirus VP1 proteins to create various nanoparticles [17,18].

Homo- and/or heterotypic interactions are also common features of the major structural proteins of other viruses, driving the self-formations of different viral capsids in nature. These features have been utilized to produce various noninfectious VLPs or viral capsid-like nanoparticles *in vitro* by expressing one or more full-length or truncated capsid proteins via various expression systems [19,20]. Such VLPs and capsid-like nanoparticles are excellent vaccine candidates against corresponding viral pathogens, because they retain arrays of antigenic epitopes that faithfully mimic those of the native virions [21], and these repeated viral antigens and epitopes stimulate strong immune responses in their animal and human hosts. In addition, such highly immunogenic subviral nanoparticles also serve as versatile platforms that are able to display foreign antigens for improved immune responses to facilitate development of novel vaccines against various pathogens and diseases.

Based on the homotypic interactions of norovirus capsid P and S domains, two subviral nanoparticles, the 24-valent P_24_ and the 60-valent S_60_ nanoparticles, as well as P domain-derived polymers, have been designed and generated through bioengineering of the two domains. These nanoparticles and polymers are easily produced, highly stable, and extremely immunogenic. The fact that these nanoparticles and polymers are composed of authentic norovirus antigens and retain norovirus-specific molecular patterns make them excellent vaccine candidates against noroviruses. In addition, the natures of self-formation, high stability, polyvalence, and high immunogenicity of the nanoparticles and polymers make them potent platforms to display foreign antigens, resulting in chimeric nanoparticles as vaccine candidates against further pathogens and diseases. Several P_24_/S_60_ nanoparticle- and polymer-based chimeric vaccine candidates have been generated and characterized, showing high protective efficacies against corresponding viral pathogens or diseases in preclinical animal studies, thus warranting their future development into useful vaccines.

## 2. Creation of Norovirus S_60_ Nanoparticles 

It has been known since the 1990s that baculovirus-expressed full-length norovirus VP1s self-assemble into VLPs [22] (Figure 1A), offering an excellent norovirus research model and a nonreplicating norovirus vaccine candidate. Previous data have also shown that heterologous expression of norovirus S domains alone via baculovirus expression system self-assembles into 180-valent S nanoparticles that are equivalent to the interior shell of norovirus capsid [23,24] (Figure 1B). However, attempts to produce such S nanoparticles via the *Escherichia coli* (*E. coli*) expression system was failed. Recently, a technology was invented to generate S nanoparticles via the *E. coli* system [25]. This was achieved through the expression of modified S domains with an R69A mutation to destroy a conserved proteinase cutting site and thus stabilize the expressed S proteins [25]. Gel filtration chromatography revealed that the majority of the S domain proteins self-assembled into the S nanoparticles. Electron microscopy (EM) showed uniform S nanoparticles about 20 nm in diameter. Finally, native mass spectrometry analysis detected 60-valent but not 180-valent S particles, indicating that these S nanoparticles were 60-valent, referred to as S_60_ nanoparticles [25]. 

### 2.1. Potential of the S_60_ Nanoparticle as a Norovirus Vaccine Candidate 

It remains unclear whether the norovirus S_60_ nanoparticle can be a vaccine candidate providing neutralization and protection against norovirus infection. The S_60_ nanoparticle consists of the S domain of norovirus VP1, which naturally forms the interior, icosahedral shell of norovirus capsids [10]. Currently, the role of the norovirus inner shell in the viral replication cycle, particularly during viral attachment and entry processes, remains unknown. A recent finding that FCV VP2s constitute a portal-like channel through the inner shell at the three-fold axis after virus–receptor engagement to deliver viral genomes to host cells during the infection process [7] suggests that the inner shell may play an important role in norovirus infection and replication. Unfortunately, a conventional cell culture-based neutralization method to determine possible neutralizing effects of the S nanoparticle-elicited antibody remains lacking. In addition, an effective small animal model for norovirus challenge to measure the protective efficacy of the S_60_ nanoparticle vaccine candidate has not yet been established. Thus, future studies are necessary to determine whether S_60_ nanoparticles can be a useful norovirus vaccine. 

### 2.2. The S_60_ Nanoparticle as a Polyvalent Platform for Antigen Display for Improved Immunogenicity

**Figure 2:**
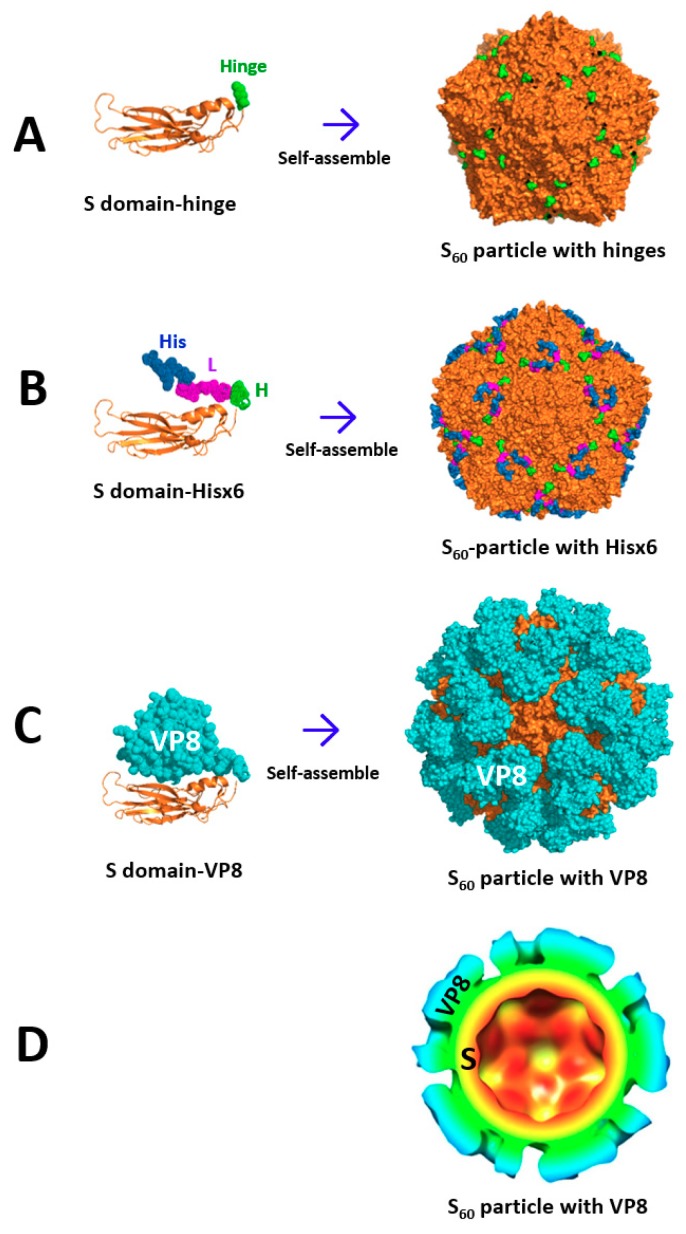
Self-formation of the norovirus S_60_ nanoparticle and its applications as a platform for epitope and antigen presentations. (**A**) Expression of the norovirus S domain (orange) with a flexible hinge (green) self-assembled into the S_60_ nanoparticle, with 60 hinges being exposed on the surface. (**B**) When the Hisx6 tag (His, dark blue) was fused to the hinge (H, green) through a linker (L, purple), the fusion proteins self-assembled into the S_60_ nanoparticle, with 60 Hisx6 tags being displayed on the surface. (**C**) When a rotavirus-neutralizing antigen VP8* (cyan) was fused to the hinge, the fusion proteins self-assembled into chimeric S_60_–VP8* nanoparticle, with 60 VP8* antigens being displayed on the surface. (**D**) Transection structure of the chimeric S_60_–VP8* nanoparticle, reconstructed by electron cryo-microscopy (cryo-EM), showing the interior S_60_ shell in red, yellow, and green, as well as the protruding VP8* antigens in cyan and green.

The structure of the S_60_ nanoparticle has been modeled using the known crystal structure of the 60-valent inner shell of FCV capsid [26]. The resulting S_60_ nanoparticle models (Figure 2A) showed pentagons at the five-fold axis and hexagons at the two-fold axis, matching the S_60_ nanoparticle shapes observed by EM [25]. Most importantly, 60 flexible hinges linked to the C-termini of the S domains were exposed on the surface of each S_60_ nanoparticle, providing excellent fusion sites for foreign antigens to be displayed on the surface of the S_60_ nanoparticles. To prove the feasibility of antigen displays by the S_60_ nanoparticles, the Hisx6 tag was fused to the C-terminus of the hinge of the S domain via a linker (Figure 2B). When the Hisx6-tagged S domain proteins were produced, the fusion proteins self-formed into S_60_ nanoparticles that could be purified efficiently using the Hisx6 tag-binding resin [25]. All approaches, including gel filtration chromatography, EM inspection, and native mass spectrometry analysis, confirmed that the Hisx6-tagged S domain proteins self-assembled into S_60_ nanoparticles [25], supporting the notion of polyvalent S_60_ nanoparticles as a potent vaccine platform.

### 2.3. Development of S_60_–VP8* Chimeric Nanoparticles as a Rotavirus Vaccine Candidate

To further prove the concept, rotavirus VP8* antigen was selected to be displayed by the S_60_ nanoparticles. Rotaviruses cause severe diarrhea in infants and young children. Even with the implementation of the current live attenuated rotavirus vaccines, the viruses still cause ~200,000 deaths, ~2.3 million hospitalizations, and ~24 million outpatient visits worldwide annually [27,28,29], suggesting a need for a new generation of rotavirus vaccines with improved efficacy. The VP8* antigen is the distal head of the rotavirus VP4 spike protein that is responsible for interacting with host glycan receptors to initiate viral infection. Therefore, VP8* is an important rotavirus-neutralizing antigen and an ideal vaccine target [30,31]. The 159-amino acid–VP8* antigen was fused to the hinge of the S domain via a linker. The resulting S–VP8* fusion proteins were easily produced through the *E. coli* expression system. Gel filtration chromatography, native mass spectrometry, and EM inspection demonstrated that the fusion proteins self-assembled into 60-valent S_60_–VP8* chimeric nanoparticles efficiently [25]. 

The three-dimensional (3D) structures of the S_60_–VP8* chimeric nanoparticles were modeled using the crystal structures of the 60-valent FCV capsid [26] (Figure 2C) and were experimentally reconstructed by cryo-EM technology (Figure 2D) [25]. The S_60_–VP8* chimeric nanoparticles revealed a *T* = 1 icosahedron consisting of 60 copies of the S–VP8* fusion proteins. The VP8* antigens formed surface protrusions extending from the interior S_60_ shell, indicating that the VP8* portions were exposed on the surface of the chimeric S_60_–VP8* nanoparticle. Fitting of the known crystal structures of the 60-valent FCV inner shell (PDB code: 4PB6) [26] and the VP8* antigens of human rotavirus Wa strain (PDB code: 2DWR) [32] to the corresponding regions of the cryo-EM electron density maps confirmed that the S_60_–VP8* nanoparticle structures reconstructed by cryo-EM were reliable [25].

The S_60_–VP8* chimeric nanoparticles were further characterized as a rotavirus vaccine candidate. A mouse immunization study showed that the VP8*-specific antibody titers elicited by the S_60_–VP8* nanoparticles with aluminum hydroxide adjuvant via intramuscular injection were significantly higher than those induced by the free VP8* antigens (*P* < 0.001), indicating that the S_60_ nanoparticles enhanced the immune response toward the displayed VP8* antigens significantly [25]. These mouse sera also exhibited significantly higher neutralization activities than the mouse sera after immunization with the free VP8* antigens (*P* < 0.01) [25]. Finally, the S_60_–VP8* nanoparticle vaccine demonstrated strong protective efficacy against rotavirus infection. This was achieved by producing and testing S–mVP8* nanoparticles displaying the VP8* antigens of murine epidemic diarrhea of infant mice (EDIM) rotavirus [33]. Mice were vaccinated by the S–mVP8 nanoparticle vaccine, followed by challenge with the homologous murine rotavirus EDIM strain and determination of rotavirus shedding dynamics in the immunized/challenged mice in comparison to those of the unimmunized controls. Virus shedding of the mice after immunization with the S–mVP8 nanoparticles was ~97% lower than that of the mock vaccinated mice or the mice vaccinated with S_60_ nanoparticles without VP8* antigens (*P* < 0.01) [33]. In conclusion, the S_60_–VP8* chimeric nanoparticle is a promising rotavirus vaccine candidate that warrants future development (Table 1).

## 3. Creation of Norovirus P_24_ Nanoparticles

The crystal structure of norovirus VLPs indicates that the P domain is involved in strong dimeric interactions forming dimeric protrusions on the viral surface [22,34,35] (Figure 1A). In addition, oligomeric interactions of the P domains are also observed at the five-fold axes to further stabilize the capsid structure [22] (Figure 1A). Indeed, when the P domain protein was expressed through the *E. coli* system, it self-assembled into P dimers [24], as well as 24-valent P nanoparticles [36], referred to as P_24_ nanoparticles (Figure 1B). It was noted that the P dimers and the P_24_ nanoparticles could exchange dynamically, depending on the concentration of the P domain protein [24], indicating that the assembled P_24_ particles at this stage were unstable and easy to disassemble back into P dimers. 

To facilitate P_24_ nanoparticle formation, inter-P domain disulfide bonds were introduced through fusion of a cysteine-containing peptide to the end of the P domain [36]. During the P_24_ nanoparticle assembly, the cysteine patches were brought to the center of the P_24_ nanoparticles, resulting in sterically close contact and thus forming inter-P domain disulfide bonds that significantly stabilized the P_24_ nanoparticles, which could no longer disassemble back into the P dimers. The 3D structures of the stabilized P_24_ nanoparticles, reconstructed by cryo-EM (Figure 1B) [37], revealed a subviral nanoparticle with a diameter of about 20 nanometers consisting of 24 P domains that organized into 12 P dimers in an octahedral symmetry. The P_24_ nanoparticles were easily produced via the bacterial system [36], were highly stable [38], and were highly immunogenic [39]. In addition, norovirus P domains could also self-assemble into 12-valent small P particles [40], referred to as P_12_ nanoparticles, through further modifications of the P domain sequences. The 3D structures of P_12_ nanoparticles have also been reconstructed by cryo-EM. 

### 3.1. The P_24_ Nanoparticle as a Norovirus Vaccine Candidate

Due to the lack of an efficient culture system to grow human noroviruses, the traditional vaccine approaches of live attenuated and inactivated virus vaccines cannot be used for norovirus vaccine development. As a result, norovirus VLPs and P_24_ nanoparticles are excellent norovirus vaccine candidates. While norovirus VLPs need a eukaryotic expression system to produce, which is time-consuming with higher costs, P_24_ nanoparticles can be produced via the *E. coli* expression system at a lower cost. As mentioned above, the P_24_ nanoparticle is composed of norovirus P domains that form the surface protrusions of authentic noroviruses to interact with viral receptors to initiate norovirus infection. Thus, the P domains are ideal vaccine targets, and the P_24_ nanoparticle is an excellent norovirus vaccine candidate. Mouse immunization studies have shown that both norovirus VLPs and P_24_ nanoparticles without adjuvant efficiently elicited innate, humoral, and cellular immunity after intranasal delivery, supporting their potentials as promising norovirus vaccine candidates [39]. In another study that evaluated and compared the P_24_ and VLP vaccines using a gnotobiotic pig norovirus challenge model [41], intranasal delivery of the P_24_ nanoparticle vaccine with monophosphoryl lipid A (MPLA) adjuvant was found to prime for stronger immune responses than VLPs did. These included significantly higher numbers of 1) activated CD4^+^ T cells in all tested tissues, 2) gamma interferon-producing (IFN-γ+) CD8^+^ T cells in the duodenum, 3) regulatory T cells (Tregs) in the blood, and 4) transforming growth factor β (TGF-β)-producing CD4^+^ CD25^-^ FoxP3^+^ Tregs in the spleen post-norovirus challenge (all *P* < 0.05). These data indicated that the P_24_ nanoparticles were more immunogenic than VLPs at the same dose [41]. In conclusion, the P_24_ nanoparticle vaccine is a promising vaccine candidate worthy of further development (Table 1).

### 3.2. The P_24_ Nanoparticle as a Platform to Display Foreign Antigens for Vaccine Development

The P_24_ nanoparticle also serves as a multifunctional platform for antigen presentation for improved immunogenicity. The outermost regions of the P_24_ nanoparticle protrusions contain surface loops that are excellent sites for foreign antigen insertion into the P_24_ nanoparticles [42,43]. As a proof of concept, the Hisx6 tag was inserted into a surface loop of the P domain through a recombinant DNA approach, followed by successful detection of the Hisx6 tag on the surface of the P_24_ nanoparticles by Hisx6-binding resins [43]. Then, a number of small peptide epitopes and large protein antigens were displayed for novel vaccine development [43].

#### 3.2.1. The P_24_ nanoparticle for Displaying Rotavirus VP8* Antigens 

**Figure 3:**
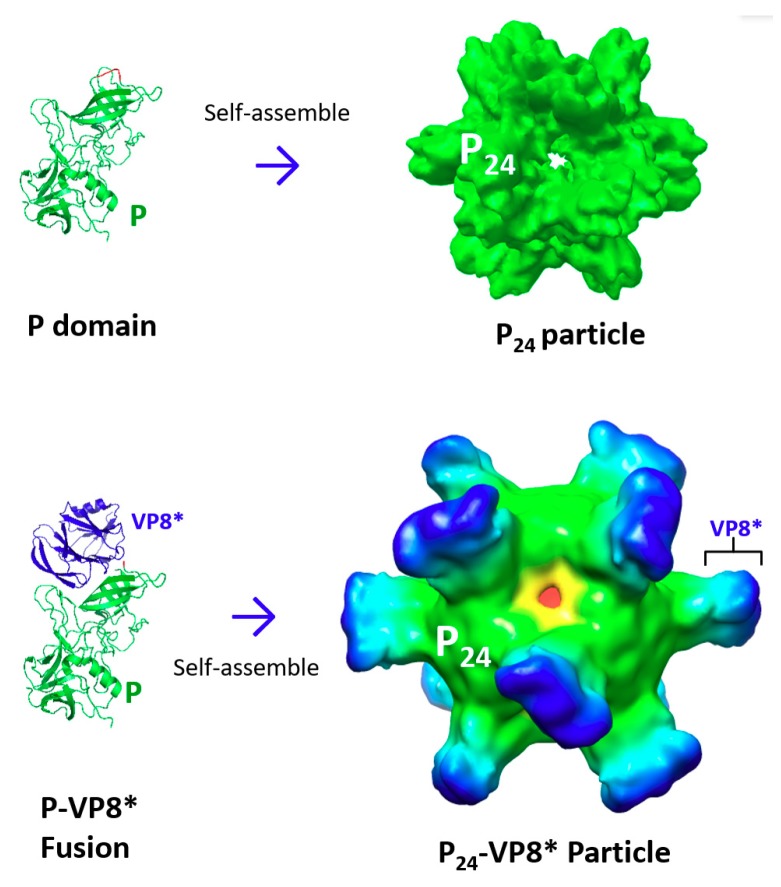
Self-formation of norovirus P_24_ nanoparticle and its application as a platform for antigen presentation. (**A**) Norovirus P domain (green) self-assembled into the P_24_ nanoparticle. (**B**) When the rotavirus-neutralizing antigen VP8* (blue) was fused to a surface loop of the norovirus P domain (green), the P–VP8* fusion proteins self-assembled into the P_24_–VP8* chimeric nanoparticle, with 24 copies of the VP8* antigens being displayed on the surface of the chimeric P_24_–VP8* nanoparticle.

The usefulness of the P_24_ nanoparticles as a polyvalent platform to display foreign antigens for vaccine development has been extensively studied through generation and characterization of a chimeric P_24_ nanoparticle displaying the rotavirus surface spike protein VP8* [43]. The 159-residue VP8* protein (see Section 2.3) of a P [8] type human rotavirus was inserted into a P domain surface loop. The resulting P–VP8* fusion proteins self-assembled into P_24_–VP8* chimeric nanoparticles with apparently larger sizes (~24 nm) than the parental P_24_ nanoparticles (Figure 3). In addition, 3D structure reconstruction of the P_24_–VP8* chimeric nanoparticles by cryo-EM, followed by fitting-in of the known crystal structures of rotavirus VP8* antigens (Wa strain, PDB code: 2DWR) [32], showed that 24 rotavirus VP8* antigens were displayed on the outermost surface of each P_24_–VP8* chimeric nanoparticle [43]. The P_24_–VP8* nanoparticles also revealed strong binding capability and specificity to the host glycan ligands of the P [8] rotavirus [44,45]. Therefore, the P_24_ nanoparticle-displayed VP8* proteins retained (most likely) their authentic structure and function.

Mouse immunization studies showed that, after intranasal delivery, the P_24_–VP8* nanoparticles without adjuvant elicited significantly higher immune responses toward the rotavirus VP8* antigen than those induced by free VP8* antigens (*P* < 0.01), while the immunogenicity to the inner P_24_ nanoparticle remained high [43]. In addition, the resulting mouse sera after immunization with the P_24_–VP8* nanoparticles exhibited significantly higher neutralizing activity against rotavirus replication in cell culture than that of the sera after vaccination with free VP8* antigens (*P* < 0.05) [43]. The mouse sera after immunization with the P_24_–VP8* nanoparticles also blocked norovirus VLP binding to noroviral glycan receptors [43]. The enzyme-linked immunosorbent assay (EIA) to measure the blocking activity of a serum sample against norovirus VLPs binding to their host receptors serves as a surrogate norovirus neutralization method [46,47] in the field due to the lack of an effective cell culture-based neutralization approach for human noroviruses [48]. Thus, P_24_–VP8* nanoparticles may be a potential dual vaccine against both rotaviruses and noroviruses (Table 1).

The application of the P_24_–VP8* nanoparticles as a rotavirus vaccine was further studied through the construction of new P_24_–VP8* nanoparticles displaying the VP8* antigens of the murine rotavirus EDIM strain, followed by determination of their protective efficacy against rotavirus infections using the murine rotavirus challenge model [43]. Rotavirus shedding of mice that were immunized with the P_24_–VP8* nanoparticles was significantly lower compared to that of the mock vaccinated mice or control mice that were vaccinated with the P_24_ nanoparticles without VP8* antigens (*P* < 0.05) [43]. Finally, in two other studies, both the P_24_ nanoparticles and the P_24_–VP8* chimeric nanoparticles were demonstrated to induce strong immune responses in vaccinated hens, resulting in high antibody titers specific to norovirus P domain and/or rotavirus VP8* antigens in both sera (IgG) and egg yolks (IgY) [49,50]. These antibodies neutralized rotavirus replication and blocked norovirus VLPs from binding to their glycan receptors. Particularly, these studies provided an efficient approach to produce large amounts of norovirus/rotavirus-specific IgYs for use as a passive immunization treatment against diarrhea caused by the two viruses. Taken together, the P_24_–VP8* nanoparticle is a promising dual vaccine against both rotaviruses and noroviruses and warrants future development.

#### 3.2.2. The P_24_ nanoparticle as a Nanoplatform to Display Epitopes of other Viral Pathogens

The P_24_ nanoparticle has also been used to display other viral epitopes for enhanced immunogenicity for novel subunit vaccine development (Table 1). These include the M2e epitope of the matrix 2 (M2) protein [51] and the HA2 protein B cell epitope [52] of influenza viruses, the B cell epitope of VP3 of enterovirus 71 (EV71) [53], and the 4E10 and 10E8 epitopes of human immunodeficiency virus type 1 (HIV-1) [54]. The M2e epitopes are the extracellular moiety of influenza virus M2 proteins. Flu caused by influenza viruses remains a deadly human disease that claims up to 500,000 lives each year [62], and vaccination has been shown to be the most cost-effective method for controlling this disease. M2 proteins form ion channels on the infected host cells [63]. Due to their high conservation in sequences among different influenza virus subtypes, M2e epitopes have been proposed to be a target for a universal flu vaccine [64]. When this 23-residue epitope was inserted into surface loops of the P_24_ nanoparticles, the resulting P–M2e fusion proteins self-assembled into P_24_–M2e nanoparticles. Mice that were immunized with the P_24_–M2e nanoparticles intranasally without adjuvant or subcutaneously with Montanide ISA720 adjuvant elicited significantly higher titers of M2e-specific antibody than that induced by mice that were immunized with free M2e epitopes (*P* < 0.001) [51]. A mouse challenge study demonstrated that the P_24_–M2e nanoparticles fully protected immunized mice from a lethal dose challenge with the H1N1 influenza virus PR8 strain [51].

Similarly, the sequences of HA2 stalk regions of influenza virus hemagglutinin (HA) are highly conserved and thus have also shown promise as targets for universal flu vaccine development [64]. When the predicted B cell epitopes of HA2 proteins (HA2:90-105) of H1 and H3 influenza A viruses and an influenza B virus were inserted into three surface loops of the P_24_ nanoparticles, chimeric P_24_ nanoparticles containing the three HA2 B cell epitopes were generated, referred to as trivalent HA2–PP [52] (Table 1). Mouse immunization studies were carried out to determine antibody response, neutralization, and protection of the chimeric nanoparticle vaccine. The results showed that 1) subcutaneous immunizations of the HA2–PP nanoparticle vaccine with Freund’s adjuvant elicited high epitope-specific IgG responses; 2) the mouse sera after immunization of the trivalent HA2–PP vaccine neutralized H3 influenza A virus and influenza B virus *in vitro*; and 3) the HA2–PP vaccine protected immunized mice from H3 influenza A virus infection [52]. These data also indicated that different epitopes could be inserted into different surface loops simultaneously for bi- or trivalent vaccine development.

The P_24_ nanoparticle has also been used to display various epitopes of EV71. EV71 causes hand, foot, and mouth disease (HFMD), which represents a serious public health concern in the Asia-Pacific region [65]. Several neutralizing B cell epitopes of EV71 have been determined as targets for vaccine development [66]. In a recent study, the P_24_ nanoparticle was utilized as a platform to measure the neutralizing and protective effects of 10 epitopes that were identified in VP1, VP2, VP3, or VP4 of EV71. Chimeric P_24_ nanoparticles displaying each of the epitopes were made by inserting the epitope into a surface loop of the norovirus P domain and expressing the P-epitope fusion proteins via the *E. coli* system, respectively [53]. Mice were then immunized with each of the resulted chimeric nanoparticles with aluminum hydroxide adjuvant intraperitoneally, followed by determination of 1) epitope-specific IgG titers of the immunized mice, 2) the neutralizing effects of the hyperimmune mouse sera against EV71 viruses in culture cells, and 3) the protective efficacies of the mouse sera against EV71 infection [53]. The results showed that the 71-6 epitope (equivalent to amino acids 176–190 of VP3) elicited the highest antibody response, and the antibody neutralized EV71 viruses in an *in vitro* microneutralization assay. In addition, incubation of the hyperimmune sera with EV71 viruses before challenge strongly reduced the infectivity of EV71 in mice [53]. These data indicated that the P_24_ chimeric nanoparticle displaying the 71-6 epitope may be a promising EV71 vaccine candidate.

A similar approach was used in an attempt to develop a nanoparticle-based vaccine against HIV-1. HIV infection harms the human immune system by destroying the white blood cells that fight infection, putting HIV-infected patients at risk for serious infections, eventually leading to acquired immunodeficiency syndrome (AIDS) [67,68]. The conserved epitopes in the membrane-proximal external region (MPER) of HIV-1 are believed to be excellent vaccine targets to elicit efficient broadly neutralizing antibodies. Fusions of the conformational 4E10 and 10E8 epitopes to the three surface loops of the norovirus P domain, followed by production of the fusion protein in *E. coli*, led to self-assembled chimeric P_24_ nanoparticles displaying each of the two epitopes on the surface, referred to as 4E10-loop123 PP or 10E8-loop123 PP (Table 1) [54]. The chimeric nanoparticles were then characterized for their immune responses in guinea pigs through subcutaneous injection with Freund’s adjuvant. The results showed high levels of MPER-binding antibodies in the sera of the chimeric nanoparticle-immunized guinea pigs. In addition, the hyperimmune sera exhibited strong neutralizing activity against several HIVs, as shown by HIV-1 envelope pseudovirus neutralization assays [54]. While further study is necessary to further prove the concept, the current outcomes suggest that chimeric P_24_ nanoparticles may offer a method to develop a new HIV vaccine.

#### 3.2.3. The P_24_ Nanoparticle as a Platform to Develop a Vaccine Against Noninfectious Disease

Alzheimer’s disease (AD) is the most common age-related neurodegenerative disorder, impacting ~46 million people around the globe [69]. Its major pathological hallmark is the accumulation of amyloid-beta (Aβ) in the brain, leading to senile plaques [70,71]. Thus, major AD treatment approaches include immunotherapies to reduce Aβ deposition in the brain [72,73,74]. An idea is to develop a recombinant vaccine targeting Aβ, producing high titers of Aβ-specific antibody to bind and inactivate Aβ [55,56]. To this end, Aβ1-6 epitopes were inserted into the three surface loops of P_24_ nanoparticles, resulting in a chimeric P_24_–Aβ1-6 nanoparticle vaccine (designated as PP-3copy-Aβ1-6-loop123) [55,56] (Table 1). The resulted chimeric nanoparticle vaccines were studied in AD mouse models via subcutaneous injection of the nanoparticle vaccines with Freund’s or CpG (TGTCGTCGTCGTTTGTCGTTTGTCGTT) adjuvant. The results showed that the P_24_–Aβ1-6 vaccine elicited high titers of Aβ42-specific antibody without T cell activation, which is a negative factor leading to autoimmune response, in a way independent from the ages of mice. Notably, the P_24_–Aβ1-6 vaccine treatment effectively decreased Aβ deposition, rescued memory loss, and repaired hippocampus damage in AD mouse models [55,56]. The Aβ-specific antibodies elicited by this immunotherapy reacted with and disrupted aggregated Aβ, reducing its toxicity. In summary, the P_24_ nanoparticle-based vaccines targeting Aβ offer a promising immunotherapeutic method against AD.

## 4. Norovirus P Domain-Derived Heterologous Polymers as Vaccines and Vaccine Platforms

The dimeric and oligomeric interactions of norovirus P domains have also been utilized to design and produce various lineage and agglomerate polymers as vaccine candidates and vaccine platforms [57,58] by taking advantage of their high valence, with repeated epitopes and antigens for improved immunogenicity. For example, when two dimeric P domains representing different norovirus genogroups or genotypes were fused together via a flexible linker, the resulting fusion proteins self-assembled into lineage polymers that displayed two P domains with distinct genetic and antigenic features. Such chimeric polymers can be used as bivalent norovirus vaccine candidates [58] (Table 1). Similarly, when the oligomeric P domain with an end-linked cysteine-containing peptide (see Section 3) was fused with dimeric glutathione-s transferase (GST), the resulted fusion proteins assembled spontaneously into agglomerate polymers as a norovirus vaccine candidate [57]. These polymer vaccine candidates have been examined by mouse immunization studies, which showed that the polymers elicited significantly stronger humoral and T cell immune responses than those induced by the monomeric or dimeric antigens (*P* < 0.01) [57,58]. These outcomes support the notion that the P domain polymers are promising norovirus vaccine candidates.

These P domain polymers also serve as platforms to display other antigens for improved immunogenicity. For instance, the M2e epitope (see Section 3.2.2) of influenza virus and the VP8* antigen of rotavirus (see Section 2.3) can be displayed by the polymers by fusing them to the norovirus P domains either to the ends or to the surface loops. The polymer formations appeared not to be affected by the inserted epitopes or antigens [57,58]. Mouse immunization experiments showed that the chimeric polymers displaying the M2e epitopes or the VP8* antigens elicited high antibody titers specific to the M2e epitopes or the VP8* antigens. Further studies showed that the sera after immunization with the chimeric polymer vaccine displaying the VP8* antigens neutralized rotavirus replication in cell culture efficiently and the M2e epitope-containing polymer vaccine fully protected immunized mice from a lethal dose challenge of an H1N1 influenza virus PR8 strain in a mouse influenza virus challenge model [57,58]. Thus, lineage and agglomerate polymers are potent vaccine platforms for epitope and antigen presentations.

Two other enterically transmitted viral pathogens, astrovirus and hepatitis E virus (HEV) that cause gastroenteritis (astrovirus) [75] or hepatitis (HEV) [76] in humans share important structural features with noroviruses. Like norovirus, both astrovirus and HEV have protruding or spike proteins on the surface that are believed to interact with host receptors for viral infection, making these protruding proteins important neutralizing antigens as vaccine targets. Interestingly, the two protruding proteins also form dimers [77,78] when they are produced in the *E. coli* system, which allows them to be components in the described polymers. For example, when the P domains of norovirus and the protruding proteins of HEV were fused together, the fusion protein assembled spontaneously into oligomers, referred to as NoV P–HEV P (Table 1) [59]. A mouse immunization study indicated that the resulting oligomers elicited high IgG titers specific to the protruding proteins of the two viruses. Of note was that the mouse sera after immunization of the oligomer vaccine intranasally without adjuvant neutralized HEV replication in a cell culture and blocked norovirus VLP binding to norovirus glycan receptors. Thus, these oligomers are a promising bivalent vaccine candidate against norovirus and HEV.

When the norovirus P domain was fused with the dimeric protruding proteins of astrovirus and HEV and the fusion proteins were produced via the *E. coli* system, the resulting fusion proteins self-assembled into oligomers [60]. These large chimeric oligomers were then tested as a trivalent vaccine candidate through preclinical animal immunization studies. Specifically, mice were immunized intranasally with the oligomer vaccine in liquid formulation with MPLA adjuvant or without any adjuvant, followed by the determinations of antibody titers specific to the three antigen components, as well as the neutralizing activities of the resulting mouse sera against two of the three viruses [60]. The results showed that the chimeric oligomer vaccine elicited significantly higher antibody titers specific to the three antigen components than those elicited by a mixture of the three separate protruding proteins [*P* < 0.05, except for the norovirus P domain (*P* = 0.067)]. The mouse sera after immunization of the oligomer vaccine also exhibited significantly higher neutralizing titers against HEV replication in a cell culture than those of the sera after immunization with the mixed antigens of the three dimeric protruding proteins (*P* < 0.05) [60]. Similarly, the blocking titer of the oligomer vaccine-immunized mouse sera against the binding of norovirus VLPs to their host glycan receptors was significantly higher than that of the mouse sera after immunization with the three mixed, dimeric protruding proteins (*P* < 0.05). However, the neutralizing titer against astrovirus has not yet been determined.

Finally, in another study, a similar trivalent oligomer vaccine that was composed of the neutralizing antigens of three enterically transmitted viruses, including the protruding proteins of astrovirus and HEV and the VP8* antigen of rotavirus, was made according to the same polymer/oligomer formation principle described above [61]. The trivalent vaccine was tested using approaches similar to the above study via a mouse model and related neutralization assays. The study outcomes showed that the oligomer vaccine elicited IgG titers specific to all three antigen components were significantly higher than those induced by a mixture of the three separate antigens (*P* < 0.01) after the vaccines were delivered intranasally with MPLA adjuvant. In addition, the mouse sera after administration of the oligomer vaccine showed a significantly higher neutralizing titer against HEVs than did the sera after vaccination of the mixed antigens of the three viruses (*P* < 0.01) [61]. As expected, the oligomer vaccine-immunized mouse sera also showed a significantly higher blocking titer against the binding of rotavirus VP8* proteins to their host glycan receptors than that of the mixed antigens-immunized mouse sera (*P* < 0.01) [61], indicating that the oligomer vaccine-immunized mouse sera could inhibit rotavirus infection [44,79]. These data support the notion that P domain-based protein polymers and oligomers are a promising platform to develop bi- and trivalent vaccine candidates against two or three viral pathogens simultaneously.

## 5. Conclusions

Through bioengineering the shell (S) and protruding (P) domains of the norovirus capsid protein, polyvalent nanoparticles and polymers/oligomers have been generated as vaccines and vaccine platforms for antigen presentation with wide applications. The S nanoparticles feature exposed and flexible hinges that offer ideal fusion sites for displaying foreign antigens. The S_60_–VP8* nanoparticle, the first application of the S nanoparticle, is a promising rotavirus vaccine. The P_24_ nanoparticle itself serves as a potent norovirus vaccine candidate because it is composed of 24 copies of norovirus-neutralizing antigens. The P_24_ nanoparticle also functions as a multifunctional platform to display antigens and epitopes of other pathogens or diseases for improved immunogenicity for vaccine development. The P_24_–VP8* chimeric nanoparticle offers a highly cost-effective vaccine candidate against childhood diarrhea diseases caused by both rotaviruses and noroviruses. P domain-based polymers/oligomers serve as multivalent vaccine candidates against enterically transmitted norovirus, HEV, astrovirus, and rotavirus. The polymers/oligomers also work as multifunctional vaccine platforms to display foreign antigens for novel vaccine development.

## Figures and Tables

**Table 1:** Summary of norovirus nanoparticles and polymers as vaccine candidates and platforms to display foreign antigens and epitopes.

Nanoparticle/Polymer	Antigen/Epitope to be Displayed (Pathogen)	Chimeric Products as Vaccine Candidate	Immunity against Pathogens or Diseases	Reference
S_60_	VP8* (rotavirus)	S_60_–VP8*	Rotavirus	[25,33]
P_24_	P domain (norovirus)	P_24_	Norovirus	[39,41]
P_24_	VP8* (rotavirus)	P_24_–VP8*	Rotavirus and norovirus	[43]
P_24_	M2e (influenza virus)	P_24_–M2e	Influenza virus	[51]
P_24_	HA2 B cell epitope (influenza virus)	Trivalent HA2-PP (P_24_-HA2:90-105)	Influenza A virus and influenza B virus	[52]
P_24_	VP3 B cell epitope (EV71)	PP-71-6 (P_24_-71-6)	EV71	[53]
P_24_	4E10/10E8 epitopes (HIV-1)	4E10-PP/10E8-PP	HIV-1	[54]
P_24_	Amyloid-beta, Aβ	PP-3copy-Aβ1-6	Alzheimer’s disease	[55,56]
P polymer	P domains (noroviruses)	NoV P_GI_-NoV P_GII_ GST NoV P^+^	Different noroviruses	[57,58]
P polymer	P domain (HEV)	NoV P-HEV P	Norovirus and HEV	[59]
P polymer	P domain (astrovirus) P domain (HEV)	Ast P-HEV P-NoV P	Norovirus, astrovirus, and HEV	[60]
P polymer	P domain (astrovirus) P domain (HEV) VP8* (rotavirus)	Ast P-HEV P-VP8*	Rotavirus, astrovirus, and HEV	[61]

Note: EV71, enterovirus 71; HIV-1, human immunodeficiency virus type 1; HEV, hepatitis E virus; Ast, astrovirus, NoV, norovirus, P, protruding domain; P^+^, the P domain with an end-linked cysteine-containing peptide that can self-assemble into oligomers; PP, P particle; GI, norovirus genogroup I; GII, norovirus genogroup II. Please see the main text for details.
